# The Prevalence of TNFα-Induced Necrosis over Apoptosis Is Determined by TAK1-RIP1 Interplay

**DOI:** 10.1371/journal.pone.0026069

**Published:** 2011-10-10

**Authors:** Seda Çöl Arslan, Claus Scheidereit

**Affiliations:** Max Delbrück Center for Molecular Medicine, Berlin, Germany; Penn State Hershey Cancer Institute, United States of America

## Abstract

Death receptor-induced programmed necrosis is regarded as a secondary death mechanism dominating only in cells that cannot properly induce caspase-dependent apoptosis. Here, we show that in cells lacking TGFβ-activated Kinase-1 (TAK1) expression, catalytically active Receptor Interacting Protein 1 (RIP1)-dependent programmed necrosis overrides apoptotic processes following Tumor Necrosis Factor-α (TNFα) stimulation and results in rapid cell death. Importantly, the activation of the caspase cascade and caspase-8-mediated RIP1 cleavage in TNFα-stimulated TAK1 deficient cells is not sufficient to prevent RIP1-dependent necrosome formation and subsequent programmed necrosis. Our results demonstrate that TAK1 acts independently of its kinase activity to prevent the premature dissociation of ubiquitinated-RIP1 from TNFα-stimulated TNF-receptor I and also to inhibit the formation of TNFα-induced necrosome complex consisting of RIP1, RIP3, FADD, caspase-8 and cFLIP_L_. The surprising prevalence of catalytically active RIP1-dependent programmed necrosis over apoptosis despite ongoing caspase activity implicates a complex regulatory mechanism governing the decision between both cell death pathways following death receptor stimulation.

## Introduction

TNFα is a pleiotropic cytokine regulating a variety of cellular responses such as proliferation, differentiation, and cell death. By binding to TNF receptor I (TNF-RI), a death receptor superfamily member, TNFα induces the formation of the plasma membrane-associated complex-I, including TRADD, TRAF2/5, cIAP1/2 and RIP1. Within complex-I, RIP1 is ubiquitinated depending on TRAF2/5 and cIAP1/2 and polybuiquitinated RIP1 (Ubi-RIP1) recruits TAK1 (TAK1/TAB2/TAB3) and IKK (IKKα/IKKβ/NEMO) kinase complexes. Then, auto-activated TAK1 phosphorylates and activates IKKβ, facilitating IKKβ-mediated IκBα degradation and downstream NF-κB activation [1], [2].

Ligation of TNF-RI by TNFα also leads to the assembly of a second, cytoplasmic multiprotein complex. Deubiquitination of RIP1 is prerequisite for the formation of complex-II comprising RIP1, RIP3, FADD and caspase-8, which is then activated by auto-cleavage and initiates apoptosis. Caspase-8 also cleaves RIP1 and inactivates it within complex-II [1], [3]. TNFα-induced apoptosis is mainly studied in the absence of *de novo* protein synthesis or in cells lacking crucial activators of the NF-κB pathway, since cells that can trigger TNFα-dependent NF-κB activation express multiple anti-apoptotic genes and block complex-II-mediated apoptosis initiation [4], [5], [6], [7].

TNFα-induced programmed necrosis, or necroptosis, is a recently defined alternative cell death pathway absolutely requiring RIP1 kinase activity and is described to dominate only when dying cells cannot activate caspase-8 [1], [3]. Under these conditions, TNFα-induced complex-II acts as the pre-necrotic ‘necrosome’ complex, where catalytically active RIP1 and RIP3 trigger rapid reactive-oxygen-species (ROS) accumulation and subsequent cell death with morphological features reminiscent of necrosis [1], [3], [8], [9], [10]. The catalytic activity of RIP1 is required for the formation of the necrosome complex [8]. Since RIP1 is inactivated by caspase-8, prevention of RIP1 cleavage by caspase inhibition is thought to be required for efficient death receptor-mediated necrosis induction.

Recent studies indicated a role for cFLIP_L_, the catalytically inactive homologue of caspase-8, in programmed necrosis inhibition [11], [12]. Hetero-dimerisation of caspase-8 and cFLIP_L_ was shown to be necessary for caspase-8 mediated protection from necrosis [12]. However, depending on its expression level, cFLIP_L_ may inhibit or augment caspase-8 activity [13], hence its mechanistic contribution in necrosis is not completely clear.

As TAK1 is a critical component of TNFα-induced NF-κB activation, cells lacking TAK1 expression undergo cell death following TNFα stimulation [14], [15], [16], [17], [18]. However, TAK1 knock-out (KO) cells are remarkably more sensitive to TNFα-induced cytotoxicity than other types of cells that cannot activate NF-κB [18], [19]. In addition, TAK1 KO mice die at an earlier embryonic stage than mice lacking IKKβ or NEMO expression [4], [5], [19], [20]. This shows that TAK1 has additional, NF-κB-independent functions during embryonic development. It has been reported that TNFα stimulation of TAK1 KO keratinocytes led to a ROS-mediated, rapid cell death [18]. Moreover, it has recently been shown that in L929 cells, a murine fibrosarcoma cell line that undergoes caspase-independent programmed necrosis upon TNFα stimulation and is commonly used as a model system for this type of cell death, down-regulation of TAK1 augmented the ongoing necrotic response [21]. Here, we dissect the molecular mechanism of TNFα-induced death of TAK1 deficient cells and show that ablation of TAK1 expression alone is sufficient for the induction of TNFα-mediated programmed necrosis in cells that are otherwise resistant to TNFα-induced cytotoxicity. We identified RIP1 as a critical mediator of TNFα-induced ROS accumulation and cell death in the absence of TAK1, indicating the induction of the necrotic pathway. TAK1 mediated the stabilization of polyubiquitinated RIP1 in complex-I and prohibited RIP1-dependent rapid necrosis, unravelling a novel functional connection between these two kinases in addition to their established cooperation in TNFα-induced NF-κB signalling. Moreover, our results demonstrated that RIP1 kinase activity-dependent cell death proceeds even in the presence of ongoing caspase activity.

## Results and Discussion

### TAK1 hinders TNFα-induced, RIP1-mediated rapid cell death

**Figure 1:**
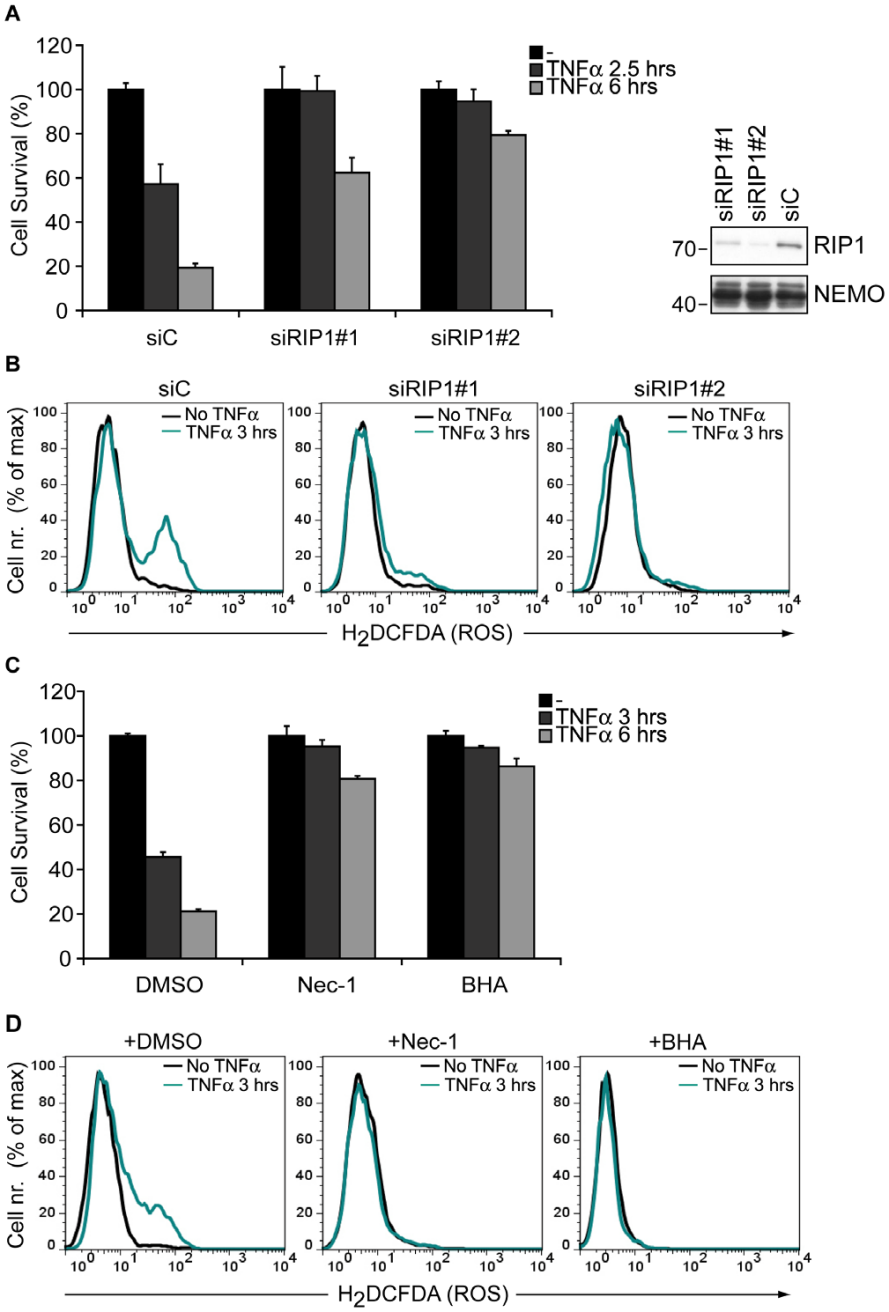
TAK1 impairs ROS-mediated cell death triggered by catalytically active RIP1. (A) *Left panel* TAK1 KO MEFs transfected with a control siRNA (siC) or with RIP1 targeting siRNAs were stimulated with TNFα and cell survival was analyzed by crystal violet staining (CV). The bar graphs depict mean values ± SEM %. *Right panel* Cell lysates were analyzed by western blotting (WB) with α-RIP1 and α-NEMO antibodies to control the efficiency of RIP1 down-regulation. (B) TAK1 KO MEFs were transfected as in (A) and stimulated with TNFα. Intracellular ROS accumulation was analyzed by flow cytometry. (C) TAK1 KO MEFs were treated with DMSO or Necrostatin-1 (Nec-1) for 1 hour and then stimulated with TNFα. Butylated hydroxyanisole (BHA) was added together with TNFα. Cell survival was analyzed by CV. The bar graphs depict mean values ± SEM %. (D) TAK1 KO MEFs were treated with DMSO, Nec-1 or BHA as in (C) and stimulated with TNFα for indicated time points. ROS accumulation was analyzed as in (B).

To evaluate whether RIP1 plays a role in the death of TAK1 KO mouse embryonic fibroblasts (MEFs), cells were transfected with two different RIP1-targeting siRNA constructs along with a control siRNA. Loss of RIP1 completely prevented TNFα-induced cell death after 2.5 hours of stimulation and provided major protection after 6 hours (Fig. 1A). In addition, TNFα-induced ROS accumulation in TAK1 KO MEFs could be fully prevented by RIP1 down-regulation (Fig. 1B). A specific RIP1 kinase- and programmed necrosis-inhibitor, Necrostatin-1 (Nec-1) [22], [23], and the ROS scavenger butylated hydroxyanisole (BHA) also completely blocked TNFα-mediated cytotoxicity (Fig. 1C) [18]. Moreover, Nec-1 was as effective as BHA in blocking TNFα-induced ROS accumulation in the absence of TAK1 (Fig. 1D). Taken together, these experiments show that catalytically active RIP1 is essential for TNFα-dependent, ROS-mediated rapid cell death in the absence of TAK1, indicating the triggering of the necrotic pathway.

### Necrosis overrules apoptosis in TNFα-treated TAK1 deficient cells

**Figure 2:**
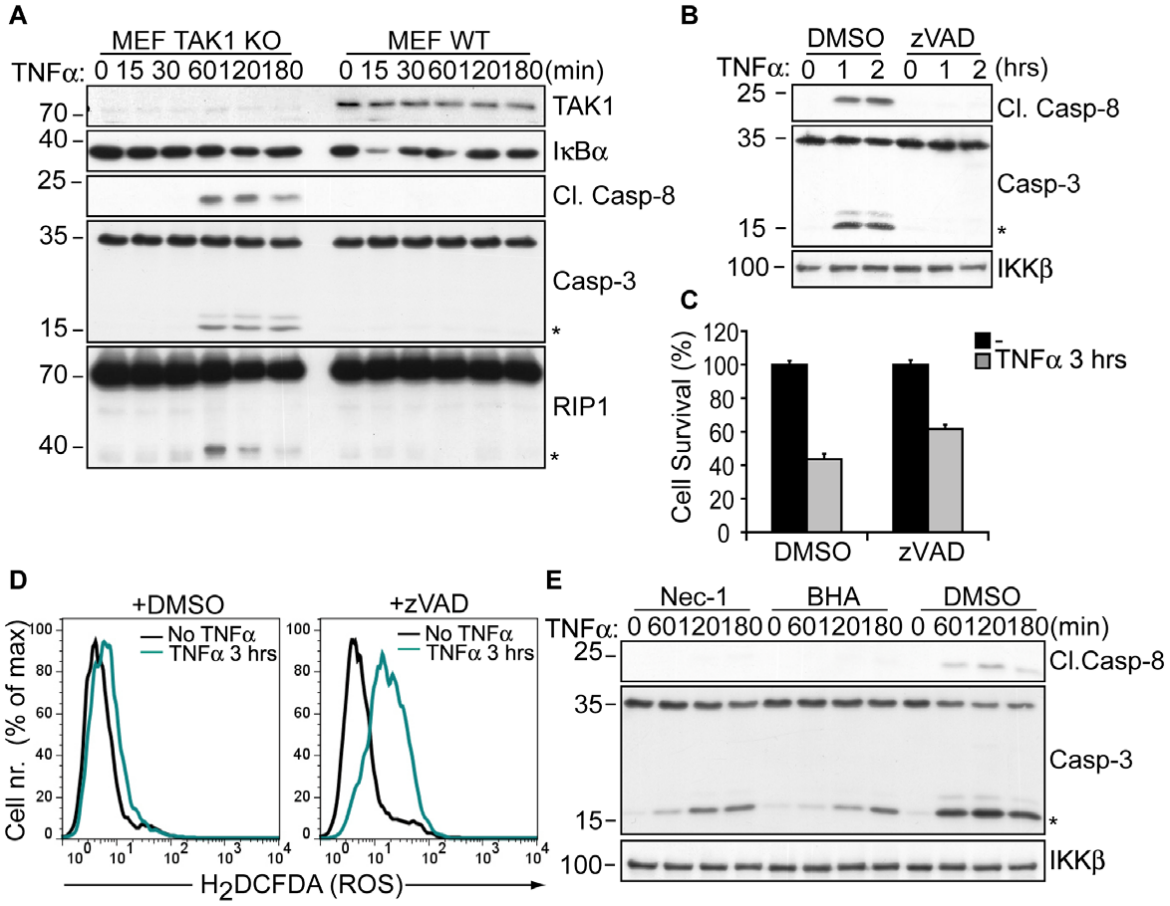
TNFα-induced caspase activity in TAK1 deficient cells is not essential for ROS-mediated cytotoxicity. (A) TAK1 KO and WT MEFs were stimulated with TNFα as indicated and analyzed by WB with the indicated antibodies to detect IκBα degradation and the cleavage of RIP1, caspase-8 (p18) and caspase-3 (p15). (B) TAK1 KO MEFs were pre-treated with DMSO or zVAD for 1 hour, stimulated with TNFα and analyzed by WB for cleaved caspase-8, caspase-3 and IKKβ. (C) TAK1 KO MEFs were pre-treated as in (B) and stimulated with TNFα. Cell survival was analyzed by CV. The bar graphs depict mean values ± SEM %. (D) TAK1 KO MEFs were pre-treated as in (B) and stimulated with TNFα. ROS accumulation was evaluated by flow cytometry. (E) TAK1 KO MEFs were pre-treated with DMSO or Nec-1 for 1 hour, stimulated with TNFα and analyzed as in (B). BHA was added together with TNFα. (*) denotes the cleaved fragments of caspase-3 (p15) and RIP1.

Death receptor-mediated programmed necrosis has been defined to predominate in the absence of caspase activity [1], [3]. However, TNFα stimulation induced the cleavage of caspase-8, caspase-3 and the caspase-8 target, RIP1 in TAK1 KO MEFs (Fig. 2A), similar to previous observations made in keratinocytes [18]. Treatment of TAK1 KO MEFs with the pan-caspase inhibitor zVAD-FMK completely abrogated caspase-8 and caspase-3 cleavage (Fig. 2B) as well as the cleavage of RIP1 and the caspase-3 target, PARP1 (Fig. S1A), nevertheless provided only minor protection from TNFα-dependent death (Fig. 2C). Moreover, zVAD failed to block TNFα-induced ROS accumulation in TAK1 KO cells (Fig. 2D). Similar observations were obtained by using another broad-spectrum caspase inhibitor, Q-VD-OPh (Fig. S1). These results indicate that TNFα-dependent caspase activation plays only a marginal role in the death of TAK1 KO cells. Of note, Nec-1 and BHA treatment attenuated caspase-8 and caspase-3 cleavage following stimulation, indicating that TNFα-induced ROS accumulation augmented caspase activation (Fig. 2E) [18]. Nec-1 and BHA provided full protection from TNFα-mediated rapid cell death without a complete block on caspase activation (Figure 1C and 2E), further supporting the conclusion that ROS-mediated necrosis is the primary cause of the rapid demise of TNFα-stimulated TAK1 KO cells, giving no time to caspase-dependent apoptotic processes to mediate cell death.

### TAK1 blocks TNFα-dependent necrosome formation

**Figure 3:**
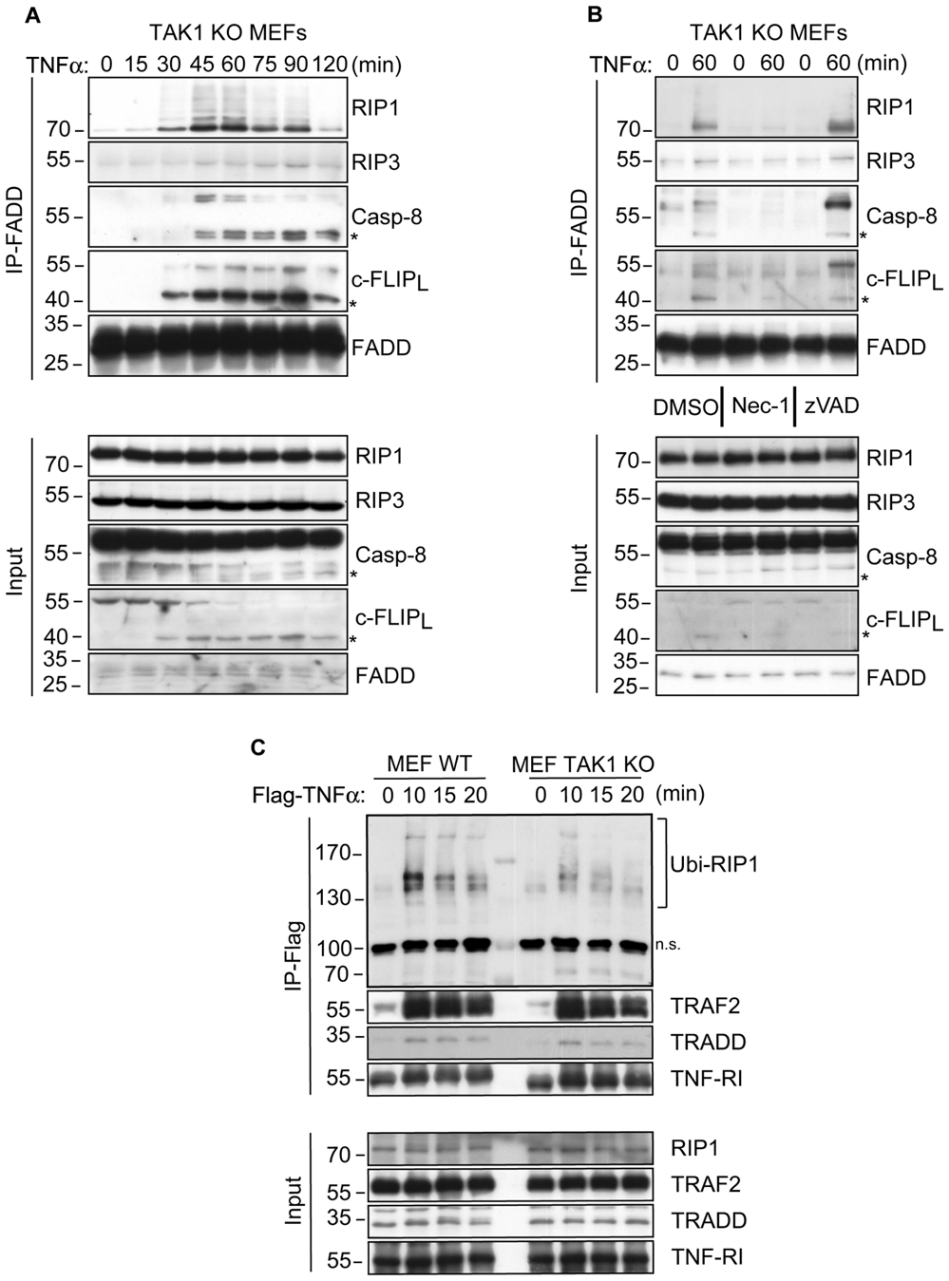
TAK1 prevents TNFα-induced necrosome formation and stabilizes polyubiquitinated RIP1 in complex-I. (A) TAK1 KO MEFs were stimulated with TNFα and lysed for immunoprecipitation with an α-FADD antibody to isolate the necrosome complex. Co-immunoprecipitated proteins were analyzed by WB with the indicated antibodies. (B) TAK1 KO MEFs were pre-treated with DMSO, Nec-1 or zVAD for 60 minutes and then stimulated with TNFα. Cell lysates were analyzed by WB as in (A). (C) WT and TAK1 KO MEFs were stimulated with Flag-TNFα for indicated time points. TNFα-bound TNF-RI and additional complex-I members were immunoprecipitated by α-Flag M2 affinity beads and analyzed by WB. Ubi-RIP1 depicts complex-I-associated polyubiquitinated RIP1. (*) denotes the p43 fragment of caspase-8 and cFLIP_L_. n.s, non-specific band.

TNFα-mediated necrotic cell death requires the formation of the necrosome complex, including RIP1, RIP3, FADD and caspase-8, whose catalytic activity has to be blocked by either small-molecule inhibitors or virally expressed anti-apoptotic proteins [1], [3]. Using FADD immunoprecipitation, we investigated whether TNFα could induce necrosome formation in the absence of TAK1. As expected, WT MEFs required co-administration of zVAD together with TNFα and CHX for efficient necrosome formation (Fig. S2). On the other hand, in TAK1 KO cells, all known components of the necrosome complex immunoprecipitated with FADD within 30-45 minutes of TNFα stimulation (Fig. 3A). Necrosome formation was fully inhibited by Nec-1 treatment, implicating an absolute dependence on catalytically active RIP1 (Fig. 3B). Thus, our study presents TAK1 as a key mediator of TNFα signalling that specifically blocks RIP1 kinase activity-dependent necrosome formation.

Surprisingly, in both WT and TAK1 deficient MEFs, the necrosome complex contained cFLIP_L_ (Fig. 3A, 3B and Fig. S2). Given its dual nature on the modulation of caspase-8 activation [13], further analysis of cFLIP_L_ within the necrosome complex may shed light on the dynamics of necrosome formation and function.

It is of note that at later time points, predominantly the cleaved forms of caspase-8 and cFLIP_L_ (43 kDa) were found in the necrosome complex, although the p43 fragment of caspase-8 was poorly identifiable in input samples (Fig. 3A and 3B). This indicated that caspase-8 in the necrosome was catalytically active and processed itself as well as cFLIP_L_. Accordingly, treatment of TAK1 KO MEFs with TNFα and zVAD enhanced the incorporation of full length caspase-8, cFLIP_L_ and RIP1 (Fig. 3B). Whether this translates into an enhanced activation of the necrosome complex is an open question, nevertheless, these experiments show that RIP1 kinase activity-dependent necrosome formation can occur in the presence of ongoing caspase activity. Also, caspase-8-mediated cleavage and the assumed inactivation of RIP1 (Fig. 2A) [1], [3] does not block programmed necrosis initiation following TNFα stimulation.

### TAK1 stabilizes Ubi-RIP1 association with complex-I

In order to analyze whether TAK1 curbs the cytotoxic potential of RIP1 by regulating its incorporation into TNFα-induced complex-I, WT and TAK1 KO MEFs were stimulated with Flag-TNFα and complex-I was immunoprecipitated with an α-Flag antibody. Stimulus-dependent incorporation of Ubi-RIP1 [7], [24] into complex-I was evident in both cell types, yet in TAK1 KO cells, the amount of Ubi-RIP1 decreased significantly at later time points compared to WT cells (Fig. 3C), indicating that TAK1 stabilizes Ubi-RIP1 in complex-I. In TNFα-treated TAK1 KO cells, the accelerated dissociation of RIP1 from complex-I was accompanied by RIP1-dependent necrosome formation within 30 minutes of stimulation. Hence, TAK1-mediated protection of cells from TNFα-induced rapid cytotoxicity might proceed via regulation of ubiquitinated-RIP1 incorporation into complex-I. In a similar manner, it has been shown that treatment of cells with Smac mimetic to diminish cIAP1/2 expression and Ubi-RIP1 association with complex-I also resulted in RIP1 kinase-activity-dependent caspase activation and subsequent cell death following TNFα stimulation [7].

### TAK1 has a kinase-independent cytoprotective function

In order to ensure that the sensitivity of TAK1 KO MEFs to TNFα-induced cytotoxicity was only due to the lack of TAK1 expression, cells were stably transfected with wild type TAK1. TAK1 KO MEFs were also reconstituted with a catalytically inactive mutant of TAK1 (TAK1-K63W [25]) to investigate whether TAK1 kinase activity was required for the protection from necrosis. By using a TAK1 kinase inhibitor (NP-009245) at 1 µM, it has been suggested that inhibition of TAK1 catalytic activity further increased caspase-independent necrosis in L929 cells [21]. However, at this concentration, NP-009245 inhibited EGF and PMA-mediated JNK phosphorylation in WT and TAK1 KO MEFs equally well, indicating that NP-009245 did not enhance TNFα-mediated necrosis specifically via TAK1 inhibition (Fig. S3).

**Figure 4:**
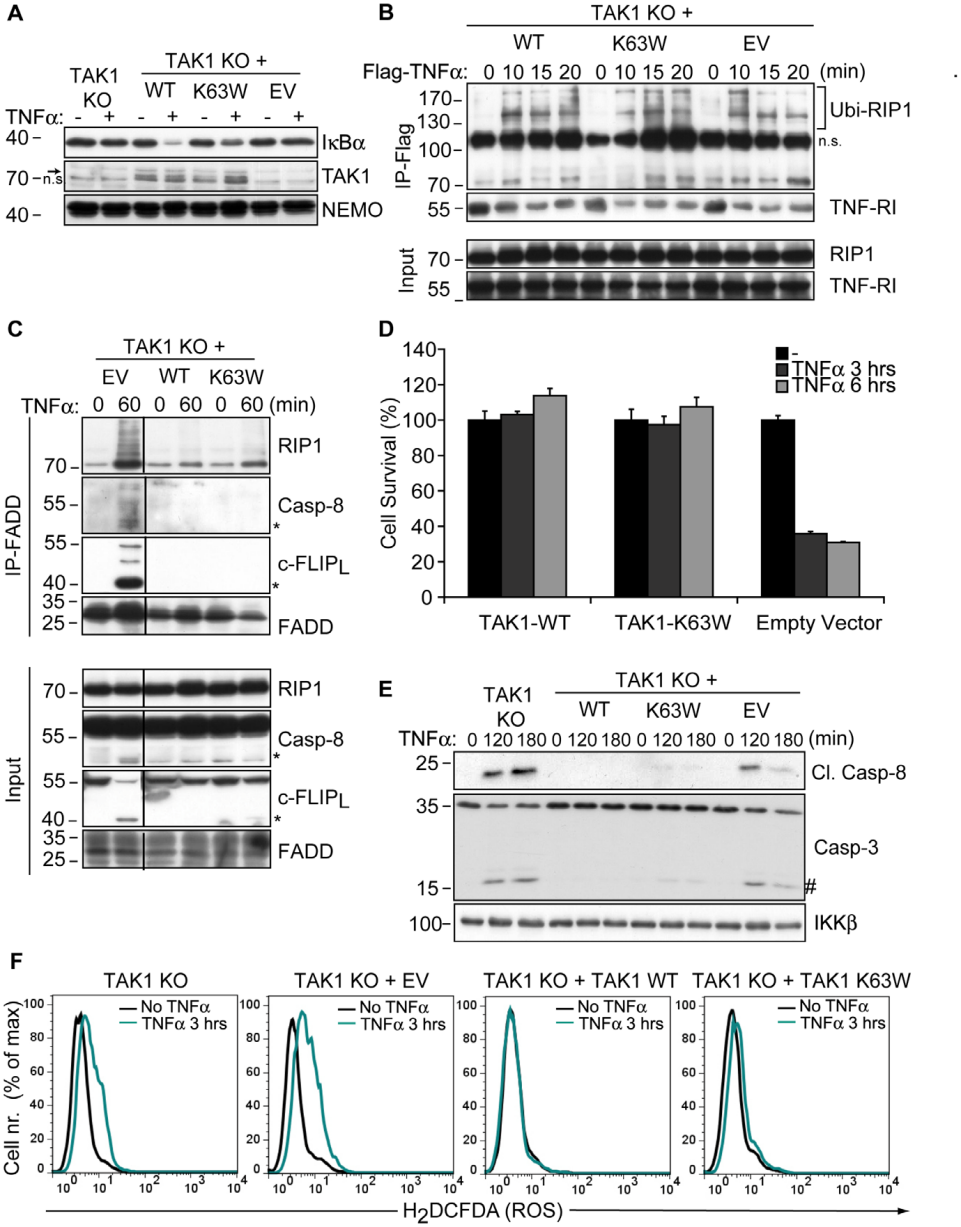
TAK1 blocks TNFα-induced necrosis independently of its kinase activity. (A) TAK1 KO MEFs transfected with a WT or a kinase-dead mutant (K63W) construct of TAK1 along with untransfected and empty-vector (EV) transfected cells were stimulated with TNFα and analyzed by WB for IκBα degradation as well as TAK1 and NEMO expression. (B) Cells were stimulated with Flag-TNFα and Ubi-RIP1 interaction with complex-I was analyzed by immunoprecipitating Flag-TNFα-bound TNF-RI and associated factors by α-Flag M2 affinity beads as in Fig. 3C. (C) Indicated cells were stimulated with TNFα and necrosome formation was investigated by immunoprecipitating FADD as in Fig. 3A and 3B. (D) Cells were stimulated with TNFα and cell survival was analyzed by CV. The bar graphs depict mean values ± SEM %. (E) Cells were stimulated with TNFα and caspase activation was assessed by WB analysis. (F) Cells were treated with TNFα and intracellular ROS accumulation was analyzed by flow cytometry. (*) denotes the p43 fragment of caspase-8 and cFLIP_L_. (#) denotes cleaved caspase-3 (p15). n.s., non-specific band.

As expected, TAK1 KO cells reconstituted with TAK1-WT were capable of TNFα-induced IκBα degradation, which was impaired in cells expressing TAK1-K63W (Fig. 4A). Nevertheless, reconstitution of TAK1 KO cells with either construct resulted in a prolonged association of Ubi-RIP1 with TNFα-induced complex-I compared to empty vector-transfected cells (Fig. 4B). This suggested a scaffold function for TAK1 to restrict the premature dissociation of RIP1 from complex-I. In parallel with this observation, TNFα-dependent necrosome formation was inhibited upon reconstitution with either TAK1-WT or TAK1-K63W (Fig. 4C). Moreover, expression of TAK1-WT or TAK1-K63W in TAK1 KO cells provided complete protection from TNFα-dependent cytotoxicity (Fig. 4D) and also blocked TNFα-induced caspase activation (Fig. 4E) as well as ROS accumulation (Fig. 4F). These data demonstrate that TAK1 acts as an adaptor molecule in complex-I to prevent premature dissociation of Ubi-RIP1 and also functions independently of its kinase activity to block TNFα-induced, RIP1-mediated necrosis. Therefore, our data suggest that TAK1 may suppress the pro-necrotic activity of RIP1 by sequestering it within complex-I, highlighting a novel non-catalytic function of TAK1 in complex-I in addition to its role as a signal transducer for TNFα-mediated NF-κB activation.

RIP1-dependent necrotic cell death is not only observed following death receptor stimulation, but also in acute pancreatitis [9], [10], ischemia-induced brain injury [23] as well as myocardial infarction [26]. Hence, future studies may reveal whether TAK1 also modulates RIP1-dependent necrosis under these pathophysiological conditions and help further characterization of TAK1-RIP1 interplay in necrotic cell death.

## Materials and Methods

### Reagents and cytokines

Recombinant human TNFα and Flag-TNFα (Alexis) were used at 20 ng/ml and 200ng/ml, respectively. Nec-1 (Biomol), BHA (Sigma), zVAD(oMe)-FMK (Imgenex) and Q-VD-OPh (Calbiochem) were utilized at 10 µM, 100 µM, 20 µM and 20 µM, respectively. TAK1-inhibitor (NP-009245) was purchased from AnalytiCon Discovery. EGF (Invitrogen) and PMA (Calbiochem) was used at 25 ng/ml and 300 ng/ml, respectively.

### Antibodies

The following antibodies were used for immunoprecipitation and western blot analyses: RIP1 (clone 38) and NEMO (clone 54) from BD Transduction, TAK1 (M-579), FADD (M-19), TRAF2 (C-20) and IκBα (C-21) from Santa Cruz Biotechnology, IKKβ (A10AG2) from Millipore, cleaved caspase-8 (#9429), caspase-3 (#9662) and p-JNK (#9251) from Cell Signaling, JNK (51-1570GR) from BD Pharmingen, caspase-8 (1G12) and c-FLIP_L_ (Dave2) from Enzo Life Sciences, RIP3 (2283) from Proscience, TNF-RI (ab 19139) and TRADD (ab 18914) from Abcam. Rabbit polyclonal antibody against PARP1 (288) was described previously [27].

### DNA and siRNA constructs

Murine TAK1-WT and K63W were subcloned into pTRACER-CMV2 (Invitrogen) from pCMV-HA-TAK1-WT and K63W constructs (gift from Dr. Kunihiro Matsumoto, Nagoya University, Nagoya, Japan) between KpnI/XbaI restriction sites. RIP1 expression was downregulated by using two different siRNA constructs (#1 sense: 5′ GCAGAGAGCUCGUGAGAAU 3′ and #2 sense: 5′ CCACUAGUCUGACUGAUGA 3′). Non-targeting control siRNA construct was 5′ UCAAAUUGGUCCACGGAUA 3′. All siRNA duplexes were purchased from Eurogentec.

### Cell culture and transfections

WT and TAK1 KO MEF cells [19] were a gift of Dr. Shankar Ghosh (Columbia University, New York, USA) and maintained in DMEM-GlutaMAX (Gibco) supplemented with 10% FCS (Gibco) and penicillin/streptomycin (100 U/ml and 100 µg/ml). To reconstitute TAK1 deficient MEFs with pTRACER-TAK1-WT and pTRACER-TAK1-K63W along with empty pTRACER for empty vector transfection, cells were transfected by using Transfectin (Biorad) reagent according to manufacturer's instructions. Stably transfected monoclonal cells were selected with 80–100 µg/ml Zeocin (Invitrogen) and expanded for experimentation. To downregulate protein expression, cells were transfected with different siRNA (10–20nM) constructs with the help of lipids from Silence Therepeutics according to manufacturer's instructions and harvested 3 days after transfection. All cells were starved overnight before analysis.

### Immunoprecipitation and western blotting

The necrosome complex was isolated as described previously [8]. Briefly, cells (∼1x10^7^) were lysed in 1 ml necrosome buffer (20 mM Tris-HCl 7.5, 150 mM NaCl, 0.2% NP-40, 1mM EDTA, 10% glycerol, 3 mM NaF, 1 mM β-glycerophosphate, 1 mM Na_3_VO_4_, 50 nM Calyculin A (Cell Signaling), 500 µM Pefablock (Roche) and protease inhibitor cocktail tablets (Roche)). Cleared lysates were incubated with α-FADD antibody for 2 hours and immunocomplexes were precipitated with protein G sepharose beads (GE Healthcare) for another hour. Co-precipitated proteins were analyzed by western blotting (WB). Standard procedures were followed for WB.

Flag-TNFα immunoprecipitation was done as described previously [24] with minor modifications. After stimulation with 200 ng/ml Flag-TNFα, cells (∼1x10^7^) were lysed in 500 µl Flag-IP buffer (30 mM Tris-HCl pH 7.5, 120 mM NaCl, 1 mM EDTA pH 8.0, 1 mM KCl, 1% Triton X-100, 8 mM β-glycerophosphate, 20 mM NaF, 200 µM Na_3_VO_4_, 500 µM Pefablock and protease inhibitor cocktail tablets). Cleared lysates were incubated with α-Flag-M2 affinity gel (Sigma) for 3 hours. For uninduced samples, 200 ng of Flag-TNFα was added to the lysates just before immunoprecipitation. Immunocomplexes were analyzed by WB.

### Intracellular ROS measurement with carboxy-H_2_DCFDA and Propidium Iodide staining

MEFs were stimulated for indicated time points and were supplemented with 10 µM carboxy-H_2_DCFDA (Invitrogen) in the last 30 minutes of incubation at 37°C. Cells were then trypsinized, washed and re-suspended in 1 ml PBS. Intracellular ROS accumulation was analyzed by using the FL1 channel of a BD FACSCalibur flow cytometer. Propidium iodide (PI, Sigma) was added (2 µg/ml) to each sample just before measurement. PI staining was visualized by using the FL3 channel. Data was analyzed by using FlowJo software. In all experiments, cells were FSC/SSC gated before analysis. ROS accumulation was visualized in the living population (PI negative cells).

### Crystal violet staining (CV)

Following medium removal, MEFs (in 12-well plates) were incubated for 5 minutes with 1 ml PFA/PBS (4%, RT). PFA/PBS was replaced by 1 ml of 0.01% CV solution (Sigma, in ddH_2_O) and incubated for 20–30 minutes. Plates were washed 3–4x with tap water and let dry briefly. CV was solubilised with methanol, and its absorbance was measured at 540 nm. All treatments were done in at least triplicates. The graphs depict mean values ± SEM %.

## Supporting Information

Figure S1 **Caspase inhibition by Q-VD-OPh does not block TNFα-induced ROS accumulation and death in TAK1 KO MEFs.** (A) TAK1 KO MEFs were pre-treated with DMSO, Q-VD-OPh (20 µM) or zVAD-FMK (20 µM) for one hour and then incubated with or without TNFα for another hour. The efficiency of caspase inhibition was assessed by WB analysis of cleaved PARP1 and RIP1. (*) denotes the cleaved products of PARP1 and RIP1 (B) TAK1 KO MEFs were pre-treated with DMSO or Q-VD-OPh (20 µM) for one hour and then stimulated with TNFα as indicated. ROS accumulation was analyzed by flow cytometry. (C) TAK1 KO cells were pre-treated as in (A) and (B), and stimulated with TNFα for 3 hours. Cells were stained with propidium iodide (PI) and analyzed by flow cytometry. The percentage of dead (PI positive) cells is indicated in each histogram.
(TIF)

Figure S2 **TNFα-induced necrosome formation in WT MEFs requires inhibition of protein synthesis and caspase activity.** WT MEFs were pre-treated for 1 hour with cycloheximide (CHX (Sigma), 1 µg/ml) and/or zVAD (20 µM) as indicated and stimulated with TNFα for 4 hours. Necrosome formation was evaluated by WB analysis with the indicated antibodies following α-FADD immunoprecipitation.
(TIF)

Figure S3 **Limited specificity of the TAK1 inhibitor NP-009245.** (A) WT and TAK1 KO MEFs were pre-treated with DMSO or NP-009245 (denoted as NP, 1 µM) for 1 hour and stimulated with EGF (25 ng/ml) or PMA (300 ng/ml) for 15 minutes. The inhibitory effect of NP-009245 was investigated by WB analysis of stimulus-dependent JNK phosphorylation, which was efficiently blocked in both TAK1 KO and WT MEFs. (B) WT MEFs were pre-treated with DMSO or increasing concentrations of NP-009245 for 1 hour and subsequently incubated with TNFα for 15 minutes. The inhibitory effect of NP-009245 on TAK1 catalytic activity was evaluated by WB analyses of IκBα degradation and JNK phosphorylation. 50 nM of NP-009245 was sufficient to block TNFα-induced JNK phosphorylation; while IκBα degradation was only blocked at 1 µM (C) WT MEFs were pre-treated as in (B) and then stimulated with TNFα for 3 hours. Cell survival was analyzed by cystal violet staining. NP-009245 induced cell death already at 250 nM. (D) WT MEFs were pre-treated as in (B) and stimulated with TNFα for 1 hour. Cells were lysed and immunoprecipitated with an α-FADD antibody to subsequently analyze RIP1 co-immunoprecipitation. Weak RIP1 co-immunoprecipitation with FADD was only observed at 1 µM of NP-009245.
(TIF)
