# Supplementary figures and images for: The Prevalence of TNFα-Induced Necrosis over Apoptosis Is Determined by TAK1-RIP1 Interplay
Source: PLoS One. 2011 Oct 10;6(10):e26069. doi: 10.1371/journal.pone.0026069 (PMC3189922; doi:10.1371/journal.pone.0026069)

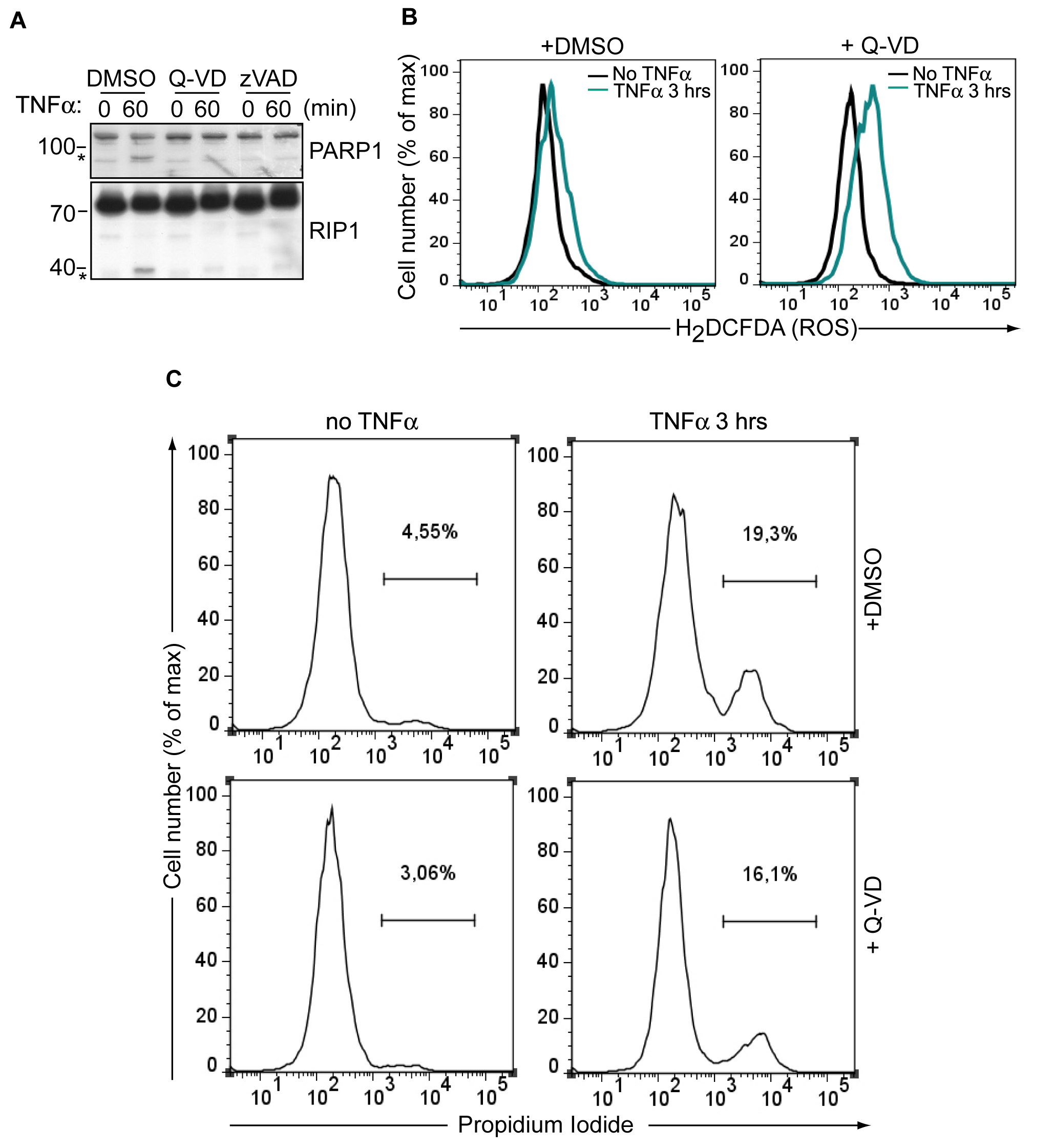

Supplement: Figure S1 — Caspase inhibition by Q-VD-OPh does not block TNFα-induced ROS accumulation and death in TAK1 KO MEFs. (A) TAK1 KO MEFs were pre-treated with DMSO, Q-VD-OPh (20 µM) or zVAD-FMK (20 µM) for one hour and then incubated with or without TNFα for another hour. The efficiency of caspase inhibition was assessed by WB analysis of cleaved PARP1 and RIP1. (*) denotes the cleaved products of PARP1 and RIP1 (B) TAK1 KO MEFs were pre-treated with DMSO or Q-VD-OPh (20 µM) for one hour and then stimulated with TNFα as indicated. ROS accumulation was analyzed by flow cytometry. (C) TAK1 KO cells were pre-treated as in (A) and (B), and stimulated with TNFα for 3 hours. Cells were stained with propidium iodide (PI) and analyzed by flow cytometry. The percentage of dead (PI positive) cells is indicated in each histogram. (TIF) [file pone.0026069.s001.tif]

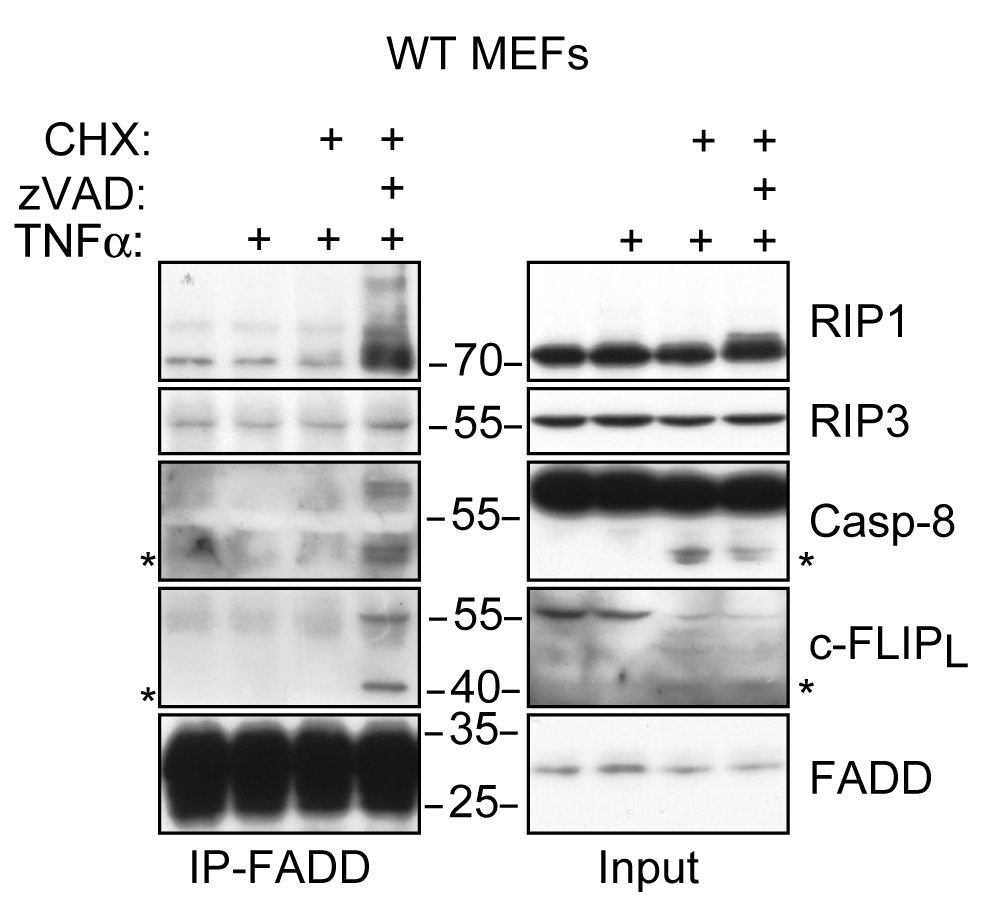

Supplement: Figure S2 — TNFα-induced necrosome formation in WT MEFs requires inhibition of protein synthesis and caspase activity. WT MEFs were pre-treated for 1 hour with cycloheximide (CHX (Sigma), 1 µg/ml) and/or zVAD (20 µM) as indicated and stimulated with TNFα for 4 hours. Necrosome formation was evaluated by WB analysis with the indicated antibodies following α-FADD immunoprecipitation. (TIF) [file pone.0026069.s002.tif]

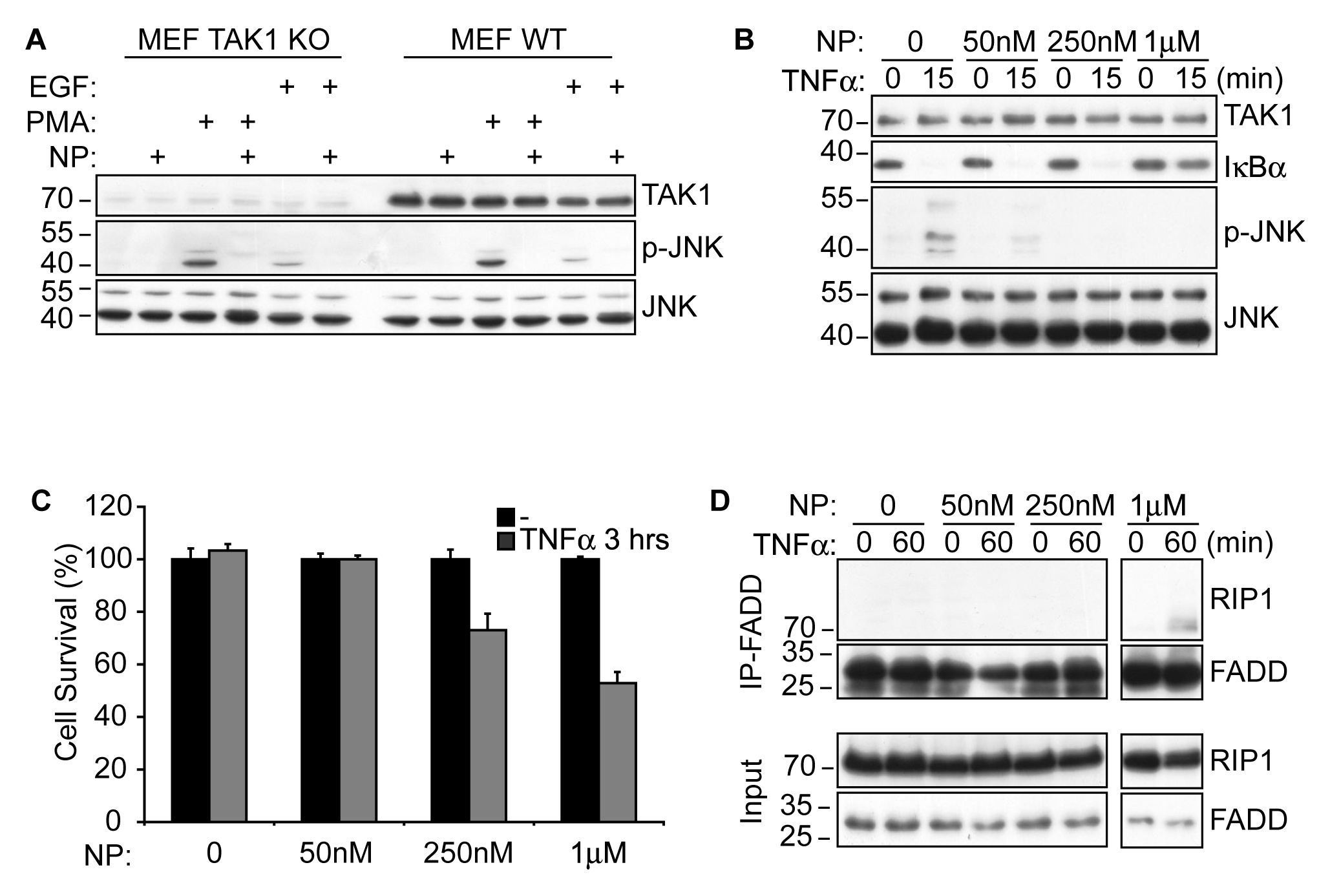

Supplement: Figure S3 — Limited specificity of the TAK1 inhibitor NP-009245. (A) WT and TAK1 KO MEFs were pre-treated with DMSO or NP-009245 (denoted as NP, 1 µM) for 1 hour and stimulated with EGF (25 ng/ml) or PMA (300 ng/ml) for 15 minutes. The inhibitory effect of NP-009245 was investigated by WB analysis of stimulus-dependent JNK phosphorylation, which was efficiently blocked in both TAK1 KO and WT MEFs. (B) WT MEFs were pre-treated with DMSO or increasing concentrations of NP-009245 for 1 hour and subsequently incubated with TNFα for 15 minutes. The inhibitory effect of NP-009245 on TAK1 catalytic activity was evaluated by WB analyses of IκBα degradation and JNK phosphorylation. 50 nM of NP-009245 was sufficient to block TNFα-induced JNK phosphorylation; while IκBα degradation was only blocked at 1 µM (C) WT MEFs were pre-treated as in (B) and then stimulated with TNFα for 3 hours. Cell survival was analyzed by cystal violet staining. NP-009245 induced cell death already at 250 nM. (D) WT MEFs were pre-treated as in (B) and stimulated with TNFα for 1 hour. Cells were lysed and immunoprecipitated with an α-FADD antibody to subsequently analyze RIP1 co-immunoprecipitation. Weak RIP1 co-immunoprecipitation with FADD was only observed at 1 µM of NP-009245. (TIF) [file pone.0026069.s003.tif]
